# Royal jelly attenuates cadmium-induced nephrotoxicity in male mice

**DOI:** 10.1038/s41598-019-42368-7

**Published:** 2019-04-09

**Authors:** Rafa S. Almeer, Gadah I. AlBasher, Saud Alarifi, Saad Alkahtani, Daoud Ali, Ahmed E. Abdel Moneim

**Affiliations:** 1 Department of Zoology, College of Science, King Saud University, Riyadh, Saudi Arabia; 2 Department of Zoology and Entomology, Faculty of Science, Helwan University, Cairo, Egypt

## Abstract

Cadmium exposure induces nephrotoxicity by mediating oxidative stress, inflammation, and apoptosis. The purpose of this study was to examine the protective effect of royal jelly on Cd-induced nephrotoxicity. Adult male mice were distributed randomly into 4 clusters: untreated, royal jelly-treated (85 mg/kg, oral), CdCl_2_-treated (6.5 mg/kg, intraperitoneal), and pretreated with royal jelly (85 mg/kg) 2 h before CdCl_2_ injection (6.5 mg/kg, intraperitoneal) for seven consecutive days. Cd concentration in the renal tissue and absolute kidney weight of the Cd-treated mice were significantly higher than those of control group. The levels of kidney function markers, kidney injury molecules-1 (KIM-1), metallothionein, lipid peroxidation, nitric oxide, tumor necrosis factor-α, interleukin-1β, and the apoptosis regulators Bax and caspases-3 also increased significantly in the renal tissue of Cd-treated mice, whereas the levels of glutathione, antioxidant enzyme activities, and the apoptosis inhibitor Bcl-2 were significantly reduced in the renal tissue of Cd-treated group. Histopathological studies showed vacuolation and congested glomeruli in the kidney tissue of Cd-treated mice. However, all aforementioned Cd-induced changes were attenuated by pretreatment with royal jelly. We therefore concluded that royal jelly attenuated Cd-induced nephrotoxicity and it is suggested that this nephroprotective effect could be linked to its ability to promote the nuclear factor erythroid 2–related factor 2 (Nrf2)/antioxidant responsive element (ARE) pathway.

## Introduction

Cadmium is a reactive metal which negatively affects mammalian organs, such as the brain, liver, kidney, placenta, and testis^1,2^. In humans, occupational and environmental exposure to cadmium cause severe degeneration to the kidney. Contaminated air, soil, drinking water, and food, as well as cigarettes are the main sources of cadmium exposure^3^. The mechanisms underlying cadmium nephrotoxicity are not fully understood. However, metallothioneins (cysteine-rich low molecular weight proteins), Cd-binding proteins containing thiol (-SH) groups, and divalent metal-ion transporter-1 are playing a pivotal role in cadmium accumulation in the kidney tissue^4^. After long-term exposure to cadmium, the glomerular filtration rate decreases significantly, which eventually leads to kidney failure^5^. Cadmium may induce nephrotoxicity by generating reactive oxygen species (ROS), inflammation, and apoptosis in the kidney tissue^1,6^. Elkhadragy *et al*.^2^ reported that S1 and S2 segments of the proximal tubules are the main target site of cadmium nephrotoxicity.

It is important to counter the cadmium toxicity-induced generation of free with natural antioxidants, as synthetic chelating agents have showed several undesirable side effects. Royal jelly is a milky secretion of the hypopharingeal and mandibular glands of worker honey bees for feeding the developing queen bees. Royal jelly consists of proteins (such as royalactin), monosaccharides, lipids, fatty acids (such as 10-hydroxy-2-decenoic acid and 10-HDA), free amino acids (eight essential amino acids valine, leucine, isoleucine, threonine, methionine, phenylalanine, tryptophan, and lysine), minerals (such as Fe, Ca, K, Na, Mg, Zn, Cu, and Mn), and vitamins (A, B complex, E, and C)^7^. The biological benefits of royal jelly include antioxidant, anti-inflammatory, anti-aging, hypoglycemic, antitumor, and hypocholesterolemic activities^8–10^. Hence, this study aimed to explore the nephroprotective activity of royal jelly (RJ) against CdCl_2_-induced nephrotoxicity in male mice.

## Material and Methods

### Chemicals

Anhydrous CdCl_2_ was procured from Sigma-Aldrich (St. Louis, MO, USA). Lyophilized royal jelly powder 6% 10-HAD, 340 mg of each is equivalent to 1000 mg crude royal jelly, was purchased from Pharco Pharmaceuticals Inc. (Alexandria, Egypt). Kidney function assay kits were all purchased from BioDiagnostic Co. (Cairo, Egypt). TRIzol reagent and PCR primers were provided by Invitrogen (Carlsbad, CA, USA). Thermo Scientific First Strand cDNA Synthesis kit was acquired from Thermo Fisher Scientific Inc. (Waltham, MA, USA). All chemicals were of high quality and analytical grade, and used without further purification.

### Dosage selection

The median lethal dose (LD50) of CdCl_2_ in mouth was 93.7 mg/kg^11^. In the present study, we have selected 6.5 mg/kg of CdCl_2_ which was 1/15 of the LD50 dose of CdCl_2_. Further, this dose of CdCl_2_ was used previously to produce nephrotoxicity in rats^2^. While, A pilot experiment was proceeded with three different doses of RJ (34, 85 and 170 mg/kg equivalent to 100, 250 and 500 crude RJs, respectively) to define the dose dependent effect of RJ in CdCl_2_-induced nephrotoxicity in mice. The pilot study reveled that RJ pretreatment at the doses of 34, 85 and 170 mg/kg markedly prevented pathological alternations in the kidney of CdCl_2_ intoxicated mice (data have not shown). However, 85 and 170 mg/kg of RJ showed the most protective effect than the other doses 34 mg/kg against Cd intoxication. Hence, we decided to choose the 85 mg/kg of RJ for our study.

### Animal treatment

Twenty-eight adult male Swiss mice (weighing 22–27 g, 10–12 weeks old) were procured from Vacsera (Cairo, Egypt). The mice were placed in the animal facility of the Zoology Department at Helwan University (Cairo, Egypt) at 22–25 °C with artificial 12-h light/dark cycle. The mice were supplied with pelleted rodent food and water *ad libitum*. For the evaluation of the nephroprotective effect of RJ on CdCl_2_-induced nephrotoxicity, mice were divided randomly into 4 groups (n = 7). Control mice received intraperitoneal (i.p.) injection of 0.9% NaCl (physiological saline) every day for 7 days. CdCl_2_ group received i.p. injection of 6.5 mg/kg CdCl_2_ each day for 7 days. RJ group was orally treated with RJ 85 mg/kg, which is equivalent to 250 crude RJs. RJ + CdCl_2_ group was orally administered with 85 mg/kg RJ 2 h prior to i.p. injection of 6.5 mg/kg CdCl_2_ daily for 7 days. CdCl_2_ was dissolved in NaCl solution. Before being sacrificed twenty-four h after the last treatment, they were housed separately for about 12 h for the collection of urine, and then the left kidney was immediately dissected, weighed, and directly homogenized in frozen 10 mM phosphate buffer (pH 7.4) to generate a 10% (w/v) homogenate for biochemical evaluation. Meanwhile, the right kidney was isolated for cadmium concentration determination and histopathological studies. All procedures were performed under anesthesia and all efforts were made to minimize suffering. All procedures involving animals were approved by the Committee of Research Ethics for Laboratory Animal Care, Department of Zoology, Faculty of Science, Helwan University (Approval No. HU2017/Z/03) in accordance with the National Institutes of Health (NIH) Guidelines for the Care and Use of Laboratory Animals, 8^th^ edition (NIH Publication No. 85–23, revised 1985).

### Concentration of Cd in the kidney tissue

The concentration of Cd in the kidney tissues were assessed using a standardized process^2^. In brief, kidney tissue samples were weighed and dried in an oven at 100 °C for 8 h. The dried samples were digested with 2 M nitric acid and 2 M hydrochloric acid at 150 °C for 5 h. The samples were diluted with deionized H_2_O to a final volume of 50 ml. Cadmium concentration was evaluated by graphite furnace atomic absorption spectrophotometry (Perkin-Elmer 3100) at 283.3 nm. Cd concentrations are expressed as µg/g of wet kidney tissue.

### Kidney function assays

The serum level of kidney function markers was measured using specific commercial kits for uric acid, urea, and creatinine according to the manufacturer’s protocols.

### Kidney injury molecule 1 assay

The renal concentration of kidney injury molecule 1 (KIM-1) was determined in kidney homogenates using enzyme-linked immunosorbent assay (ELISA) kit obtained from Abcam (Catalogue No. ab119597; Cambridge, UK). The assay procedures performed according to the ELISA kit instructions.

### Metallothionein determination

Metallothionein level in the kidney homogenates were measured according to the methods of Linde and Garcia-Vazquez^12^. Briefly, the homogenates were centrifuged at 30,000 × *g* for 20 min to obtain a supernatant containing metallothionein. 1.05 ml of cold (−20 °C) absolute ethanol and 80 μl of chloroform per 1 ml of the resulting supernatant were added. The cold samples (at 0–4 °C) were centrifuged at 6000 × *g* for 10 min. 3 volumes of cold ethanol were added to the resulting supernatant and store at −20 °C for 1 h, and then, the samples were centrifuged at 6000 × *g* for 10 min. The resulting pellets were washed with ethanol:chloroform:homogenization buffer (87:1:12) and then were centrifuged again at 6000 × *g* for 10 min. The dried pellet was resuspended in 300 μl of 5 mM Tris‐HCl, 1 mM EDTA, pH 7. The resuspended metallothionein fraction was added to 4.2 ml of 0.43 mM 5,5′‐dithiobis(nitrobenzoic acid) in 0.2 M phosphate buffer, pH 8. After 30 min, the concentration of reduced sulfhydryl was determined by reading the absorbance at 412 nm in a spectrophotometer. The amount of metallothionein in the samples was determined from the equation x = (2.5–0.0524)/5.5553 = μmol.

### Biochemical assays

Lipid peroxidation (LPO) was assessed as thiobarbituric acid reactive substances (TBARS) in terms of formed malondialdehyde (MDA) according to a method described by Ohkawa *et al*.^13^. Nitric oxide (NO) level was determined using Griess reagent^14^. Glutathione (GSH) content was assessed as according to a method illustrated by Ellman^15^. Superoxide dismutase (SOD) activity was valued using the nitroblue tetrazolium (NBT) method as defined by Nishikimi *et al*.^16^. Catalase (CAT) activity was determined according to a method by Aebi^17^, Glutathione peroxidase (GSH-Px) activity was determined utilizing the method of Paglia and Valentine^18^. Glutathione reductase (GSH-R) activity was analyzed utilizing the process described by De Vega *et al*.^19^.

### Inflammation marker assays

Renal levels of tumor necrosis factor-α (TNF-α; Cat. No. EZMTNFA, Millipore) and interleukin-1β (IL-1β; Cat. No. EM2IL1B, ThermoFisher Scientific) were measured using kits according to the manufacturer’s protocols.

### Western blot analysis

We performed protein extraction and western blot analyses as described previously^20^. The utilized antibodies included mouse antibody to NQO1 (sc-376023, 1:500; Santa Cruz Biotechnology, Santa Cruz, CA, USA), Nrf2 (MAB3925, 1:500; R&D System), NFκB (p65) (sc-8008, 1:200; Santa Cruz Biotechnology), goat antibody to HO-1 (AF3169, 1:500; R&D System), β-actin (MAB8929, 1:500; R&D System), goat anti-mouse IgG (sc-2039, 1:5,000; Santa Cruz Biotechnology, Santa Cruz, CA, USA) and donkey anti-goat IgG (sc-2042, 1:5,000; Santa Cruz Biotechnology, Santa Cruz, CA, USA). The proteins were visualized using an enhanced chemiluminescence detection kit (Bio-Rad, USA) following the manufacturer’s protocol. Images were analyzed using the Kodak Image Station 2000R (Eastman Kodak Company, Rochester, NY, USA). Protein bands intensity were normalized to β-actin, and the data expressed in terms of percent relative to controls.

### Quantitative RT-PCR

Total RNA was isolated from the kidney tissues using TRIzol reagent according to the instructions of the manufacturer. Approximately 5 µg of the total RNA were reverse transcribed to synthesis cDNA using a cDNA synthesis kit according to the manufacturer’s protocol. The sense/antisense primers for tested genes are listed in Supplementary Table 1. Real-time PCR analysis was performed using Power SYBR^®^ Green Master Mix kit. The relative gene expression of *Sod2*, *Cat*, *Gpx1*, *Gsr*, *Nfe2l2*, *Nos2*, *Il1β*, *Tnf*, *Bcl2*, *Bax* and *Casp3* was determined and expressed as proportional changes with respect to the control. The housekeeping gene glyceraldehyde-3-phosphate dehydrogenase (*Gapdh*) was employed as the internal control and was shown to be unaffected by the treatments.

### Histological examination

Right kidney samples were fixed in 10% neutral formalin for 24 h at 25 °C, embedded in paraffin, and sliced into 4–5 μm–thick sections. The sections were stained with hematoxylin and eosin (H&E) for light microscopy. Nikon Eclipse E200-LED (Tokyo, Japan) was used to take images of the specimens with 400× magnification.

### Immunohistochemistry analysis

For the detection of apoptosis-related proteins, the prepared kidney sections (4 μm thick) were blocked with 0.1% hydrogen peroxide containing methanol for 15 min to terminate endogenous peroxidase activity. Afterwards, the tissue slices were incubated with rabbit polyclonal Bcl-2, Bax, or caspase-3 antibody at 4 °C for 24 h. The tissues were washed with phosphate-buffered saline, incubated with biotinylated goat anti-rabbit immunoglobulins, and incubated with streptavidin–peroxidase complexes at 30 °C for 30 min. The peroxidase activity was developed with diaminobenzidine (DAB)-hydrogen peroxide. Nikon Eclipse E200-LED (Tokyo, Japan) was used to take microscopy images of the specimens at 400× magnification.

In the immunohistochemically-stained tissues, the color intensity of each protein was semi-quantitatively evaluated. The intensity was expressed as + (weak immunoreactivity), ++ (moderate immunoreactivity), +++ (strong immunoreactivity), or ++++ (very strong immunoreactivity).

### Statistical analysis

One-way analysis of variance (ANOVA) was used for the statistical analysis with *post-hoc* Tukey’s test. Results are expressed as the mean ± SD (standard deviation). Differences were considered statistically significant at *P* values < 0.05.

## Results

Mice intoxicated with Cd showed some clinical signs of cadmium toxicity including inappetence, increase in urination, slight decrease of the body weight and increase in respiratory (data not shown). However, mice pretreated with RJ showed less or no clinical signs of Cd toxicity.

**Figure 1:**
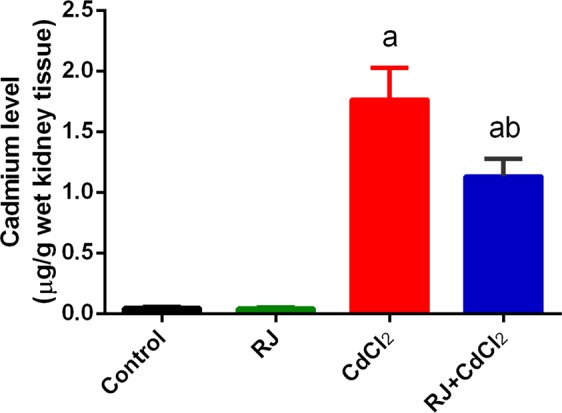
Effects of royal jelly (RJ) on cadmium concentration in the kidney of CdCl_2_-treated mice. Data are expressed as the mean ± SD (n = 7). ^a^*p* < 0.05 *vs*. the control mice; ^b^*p* < 0.05 *vs*. the CdCl_2_-treated mice analyzed using Tukey’s test.

**Figure 2:**
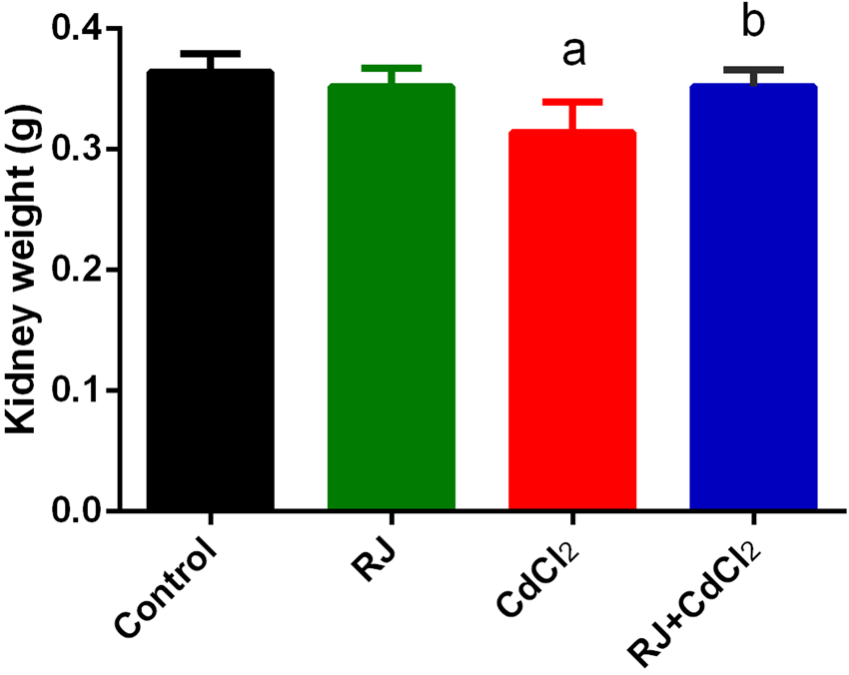
Effects of royal jelly (RJ) on absolute kidney weight in CdCl_2_-treated mice. Data are expressed as the mean ± SD (n = 7). ^a^*p* < 0.05 *vs*. the control mice; ^b^*p* < 0.05 *vs*. the CdCl_2_-treated mice analyzed using Tukey’s test.

**Figure 3:**
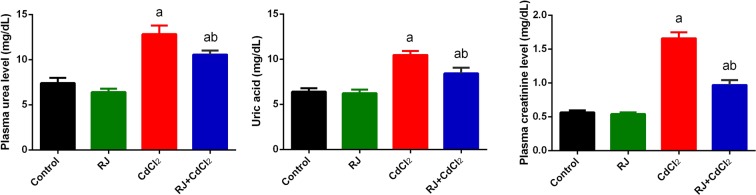
Effects of royal jelly (RJ) on the plasma level of kidney function markers in CdCl_2_-treated mice. Data are expressed as the mean ± SD (n = 7). ^a^*p* < 0.05 *vs*. the control mice; ^b^*p* < 0.05 *vs*. the CdCl_2_-treated mice analyzed using Tukey’s test.

Mice treated with CdCl_2_ showed a significant increase (*P* < 0.05) in Cd concentration in the kidney tissue compared with that of control group (Fig. 1). The daily injection of CdCl_2_ for 7 days caused significant decrease in absolute kidney weight compared with that of the control (Fig. 2). Furthermore, Cd accumulated in the kidney was concomitant with kidney function impairment, as indicated by the significant increase in creatinine, urea, and uric acid levels in Cd-injected mice (Fig. 3). RJ pretreatment abrogated all of those deterioration effects of Cd, as suggested by the significant decrease in Cd concentration, level of kidney function markers, and kidney weight compared with those of CdCl_2_-treated mice. No change in kidney weight and level of kidney function markers occurred in mice that were treated with RJ alone.

**Figure 4:**
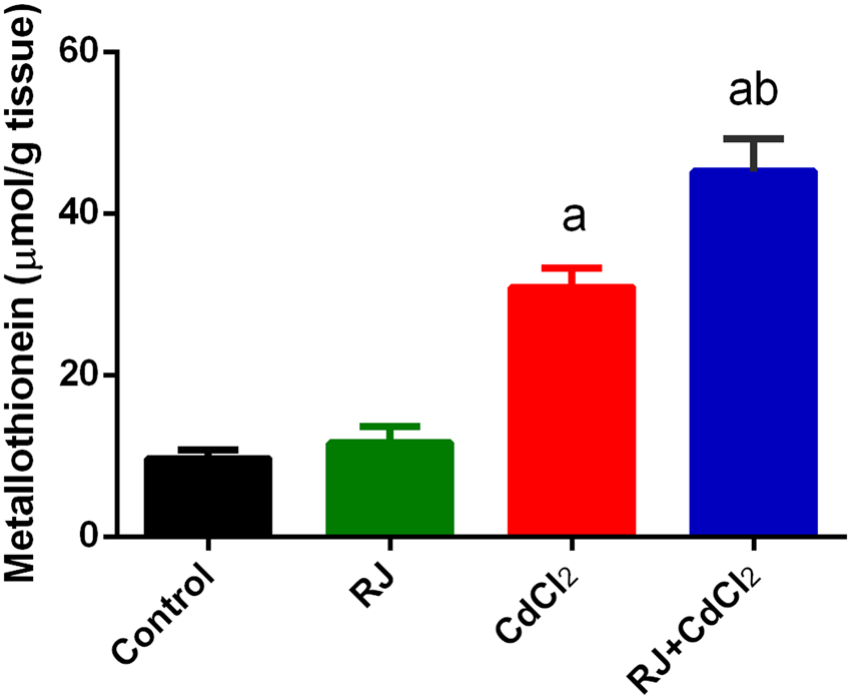
Effects of royal jelly (RJ) on the level of kidney metallothionein in CdCl_2_-treated mice. Data are expressed as the mean ± SD (n = 7). ^a^*p* < 0.05 *vs*. the control mice; ^b^*p* < 0.05 *vs*. the CdCl_2_-treated mice analyzed using Tukey’s test.

**Figure 5:**
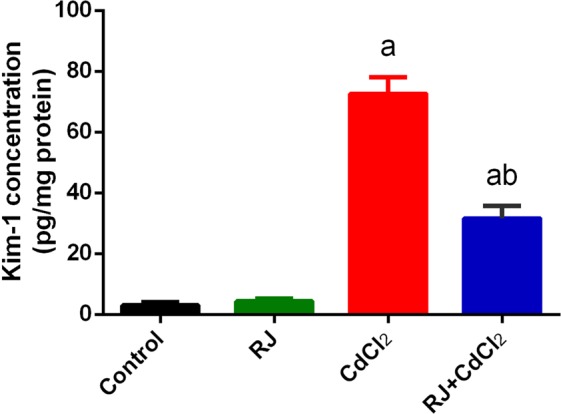
Effects of royal jelly (RJ) on the concentration of kidney injury molecule 1 in CdCl_2_-treated mice. Data are expressed as the mean ± SD (n = 7). ^a^*p* < 0.05 *vs*. the control mice; ^b^*p* < 0.05 *vs*. the CdCl_2_-treated mice analyzed using Tukey’s test.

Metallothionein (MT) is characterized by their ability to bind metals such as Cd. Hence, the concentration of MT was determined in the present study, MT concentration in kidney of rats was shown in Fig. 4. The MT concentration was significantly increased in kidney of rats those exposed to Cd. On the other hand, RJ pretreatment minimized this increment in MT. however, compared with the control; MT concentration was also significantly increased in kidney tissue. As a result of MT induction, Cd was slowly released from the kidney and that causes nephrotoxicity evidenced by the significant increase in KIM-1 in kidney of the exposed rats (Fig. 5). However, RJ administration was significantly decreased KIM-1 concentration.

**Figure 6:**
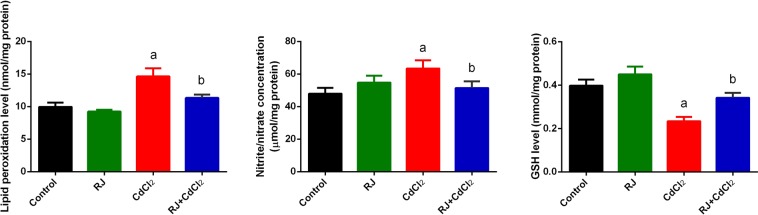
Effects of royal jelly (RJ) on the LPO, NO, and GSH levels in the kidney of CdCl_2_-treated mice. Data are expressed as the mean ± SD (n = 7). LPO, lipid peroxidation; NO, nitric oxide; GSH, glutathione. ^a^*p* < 0.05 *vs*. the control mice; ^b^*p* < 0.05 *vs*. the CdCl_2_-treated mice analyzed using Tukey’s test.

A drastic increase (*P* < 0.05) in NO along with LPO levels, coupled with a major reduction in GSH content, were observed in the kidney tissue of CdCl_2-_treated mice. RJ administration prior to CdCl_2_-treatment markedly restored the level of oxidative stress markers near that of the control group (Fig. 6).

**Figure 7:**
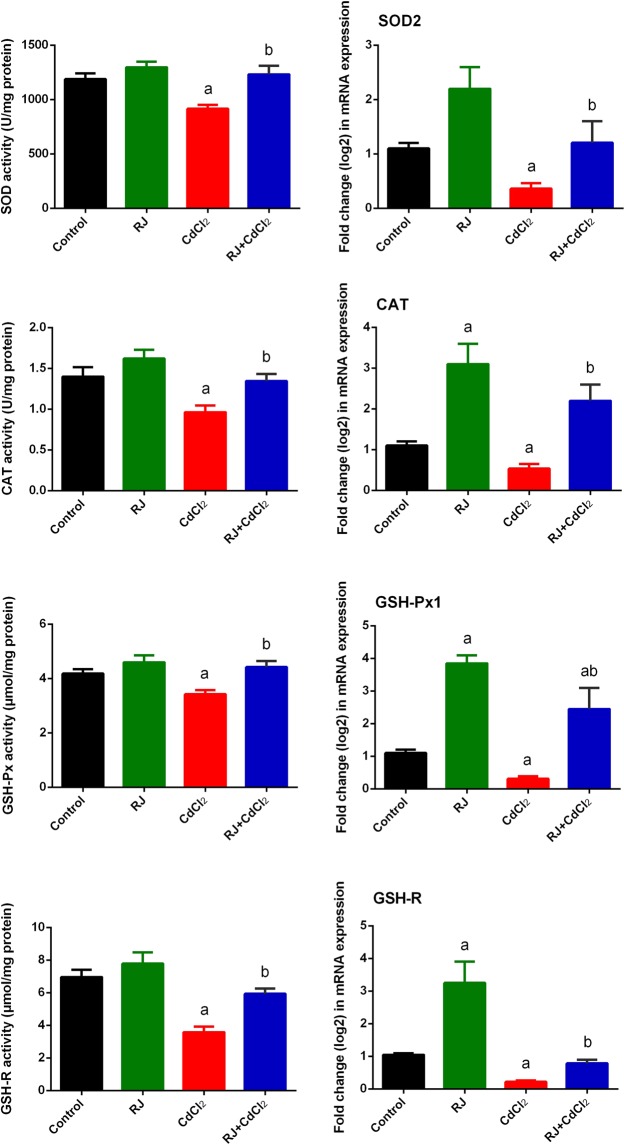
Effects of royal jelly (RJ) on the enzymatic activity and expression of SOD, CAT, GSH-Px, and GSH-R in the kidney of CdCl_2_-treated mice. Antioxidant activities are expressed as the mean ± SD of 7 mice, whereas mRNA expression are expressed as the mean ± SD of triplicate assays and were normalized to GAPDH and expressed as fold induction (log 2 scale) relative to mRNA level in the control group. SOD_2_, superoxide dismutase 2; CAT, catalase; GSH-Px1, glutathione peroxidase 1; GSH-R, glutathione reductase; GAPDH, glyceraldehyde 3-phosphate dehydrogenase. ^a^*p* < 0.05 *vs*. the control mice; ^b^*p* < 0.05 *vs*. the CdCl_2_-treated mice analyzed using Tukey’s test.

**Figure 8:**
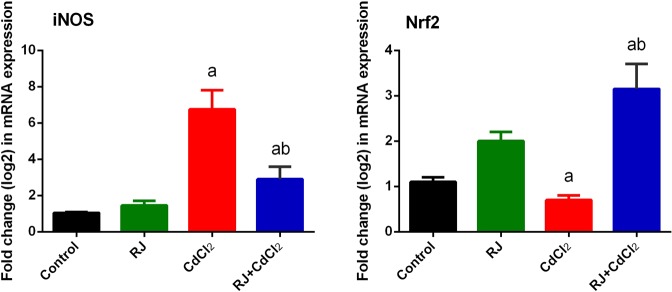
Effects of royal jelly (RJ) on the gene expressions of Nrf2 and iNOS in the kidney of CdCl_2_-treated mice. Data (mean ± SD of triplicate assays) were normalized to those of GAPDH and expressed as fold induction (log 2 scale), relative to the mRNA level in the control. ^a^*p* < 0.05 *vs*. the control mice; ^b^*p* < 0.05 *vs*. the CdCl_2_-treated mice analyzed using Tukey’s test.

**Figure 9:**
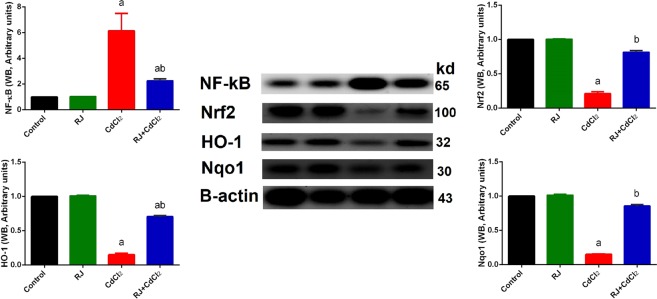
Western blot (WB) analysis and densitometric quantification of NF-κB and Nrf2 and its putative target proteins HO-1 and NQO1 in the kidney of mice intoxicated with CdCl_2_ and pretreated with royal jelly (RJ). Data (mean ± SD of triplicate assays). β-actin was used as the loading control. Molecular weight of proteins is indicated at the right-hand side. Full-length blots/gels are presented in Supplementary Fig. S1. ^a^*p* < 0.05 *vs*. the control mice; ^b^*p* < 0.05 *vs*. the CdCl_2_-treated mice analyzed using Tukey’s test.

A major inhibition (*P* < 0.05) in enzymatic activity of antioxidants (SOD, CAT, GSH-Px, and GSH-R) was noticed in mice injected with CdCl_2_ (Fig. 7). The pretreatment with RJ along prior to CdCl_2_ injection ameliorated the adverse effects of Cd, as evidenced with the restoration of enzymatic activity of antioxidants. Consistent with these biochemical findings, qRT-PCR result revealed that mRNA expression of *Sod2*, *Gpx1*, *Cat*, and *Gsr* were prominently downregulated in CdCl_2_-treated mice and RJ pretreatment upregulated these genes (Fig. 7).Nuclear factor (erythroid-derived 2)-like 2 factor is the master regulator of antioxidant protein expression in the cell which protects it from oxidative damage triggered by injury and inflammation, whereas inducible nitric oxide synthase (iNOS) is responsible for producing large quantities of NO. Thus, high level of NO have increase the chance of it reacting with oxygen free radicals, which may lead to peroxynitrite formation and subsequently cell toxicity^21^. In the kidney of Cd-treated mice, the mRNA expression of *Nfe2l2* was downregulated, whereas *Nos2* expression was significantly upregulated (Fig. 8). However, RJ pretreatment alleviated the adverse effect of Cd. Taken together; the qRT-PCR results suggested a protective effect of RJ against Cd-induced oxidative stress. The study also examined nuclear factor kappa B (NF-κB) and Nrf2 and the expression of its down-stream target genes heme oxygenase 1 (HO-1) and NAD(P)H quinone oxidoreductase 1 (NQO1). Cd exposure in rats induced a significant increase in NF-κB and a significant decrease in Nrf2 and its putative target genes, compared to the control group. However, RJ pretreatment abolished the Cd-induced impairment in the cellular detoxification system by enhancing Nrf2, HO-1, and NQO1 protein expressions and restraining NF-κB protein expression (Fig. 9).

**Figure 10:**
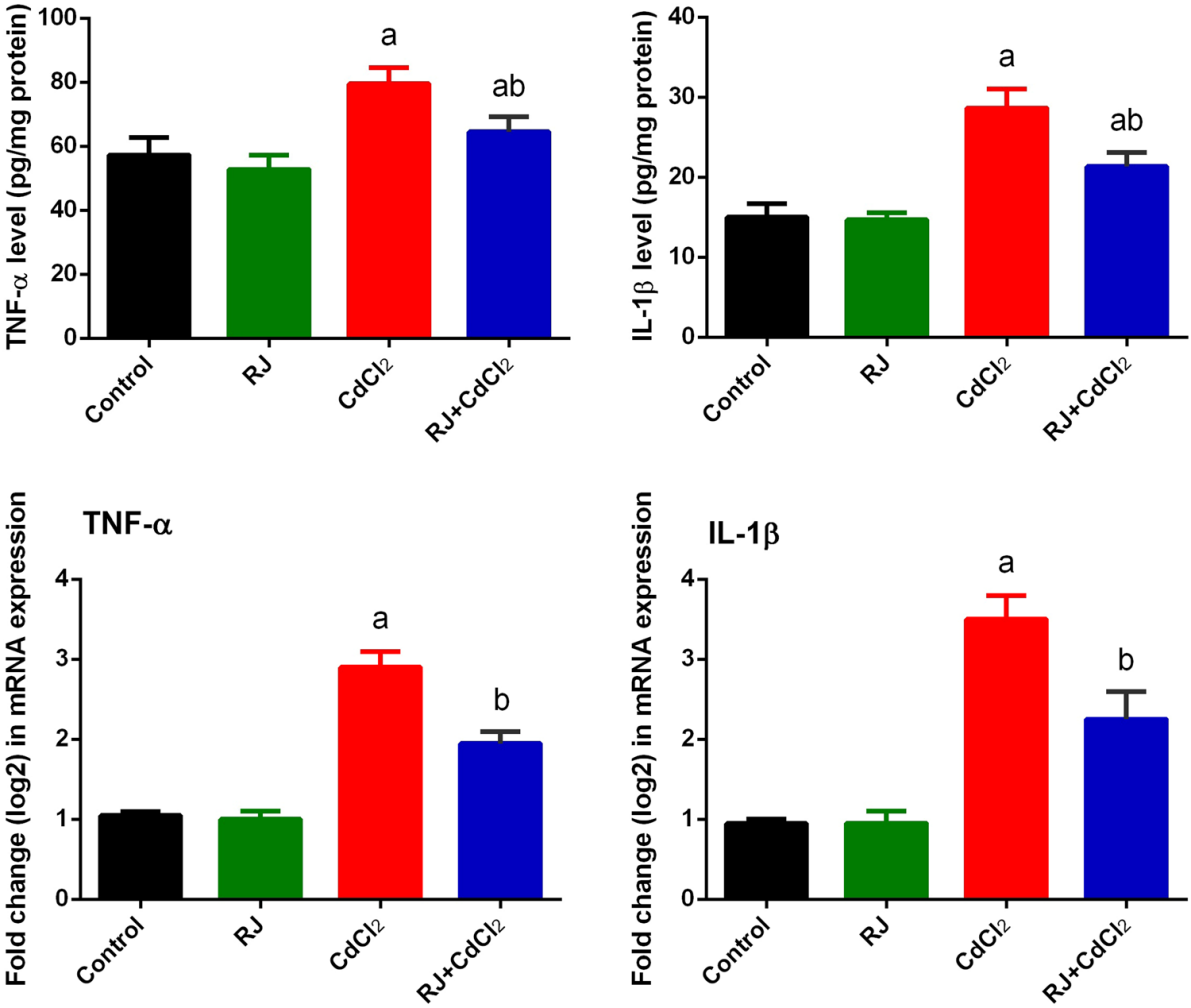
Effects of royal jelly (RJ) on the levels and gene expression of TNF-α and IL-1β in the kidney of CdCl_2_-treated mice. Data of ELISA findings are expressed as the mean ± SD of 7 mice, whereas data of mRNA expression (mean ± SD of triplicate assays) were normalized to those of GAPDH and expressed as fold induction (log 2 scale), relative to the mRNA level in the control. TNF-α, tumor necrosis factor-α; IL-1β, interleukin 1β. ^a^*p* < 0.05 *vs*. the control mice; ^b^*p* < 0.05 *vs*. the CdCl_2_-treated mice analyzed using Tukey’s test.

The kidney levels of TNF-α and IL-1β were drastically (*P* < 0.05) increased in Cd-intoxicated mice in comparison with the untreated mice. RJ pretreatment substantially (*P* < 0.05) decreased the kidney levels of both TNF-α and IL-1β, compared with those of CdCl_2_ alone-treated mice (Fig. 10). In consistence with ELISA findings, qRT-PCR findings revealed that mRNA expressions of *Tnf* and *Il1β* were significantly upregulated in the kidney of mice treated with CdCl_2_. However, RJ pretreatment was able to downregulate these genes.

**Figure 11:**
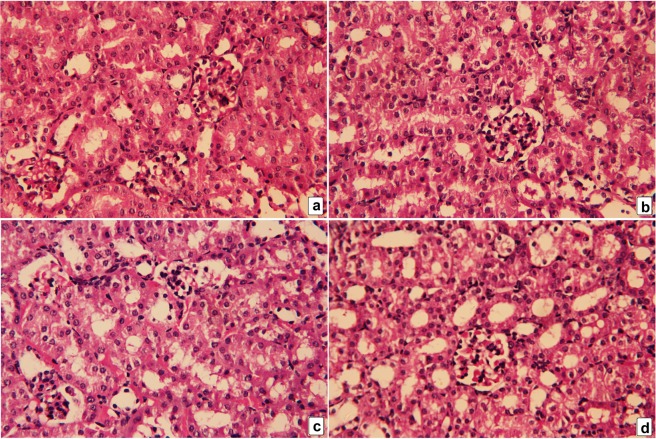
Photomicrographs of mice kidney. Kidney tissues from the control and royal jelly-treated groups (**a**,**b**, respectively) shows normal kidney structure. In the cadmium chloride (CdCl_2_)-treated mice (**c**), severe inflammation, cytoplasmic vacuolation, severe tubular necrosis and apoptosis, and congested glomeruli are shown. Pretreatment with royal jelly (**d**) markedly attenuated all renal damages caused by cadmium. Hematoxylin and eosin (H&E), 400× magnification.

The kidney tissues of control mice and RJ alone-treated mice showed normal morphology of glomeruli, proximal, and distal tubular cells (Fig. 11a,b, respectively). Mice injected with CdCl_2_ exhibited obvious damage with congested glomeruli, severe inflammatory leukocytes infiltration, cytoplasmic vacuolation, and tubular degeneration and desquamation (Fig. 11c). Several nuclei of the tubular epithelial cells were in various degrees of enlargement, the other nuclei were pyknotic. In contrast, the kidney of mice pretreated with RJ showed significant improvements in the kidney architecture (Fig. 11d).

**Figure 12:**
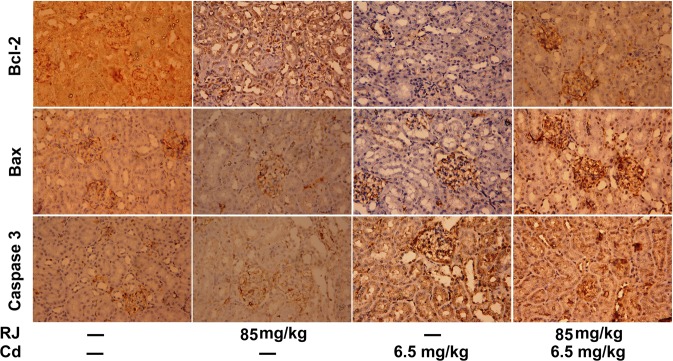
Photomicrographs of changes in Bcl-2, Bax, and caspase 3 expressions in mice kidney tissue following royal jelly (RJ) and cadmium chloride (CdCl_2_) treatments (400× magnification)

**Figure 13:**
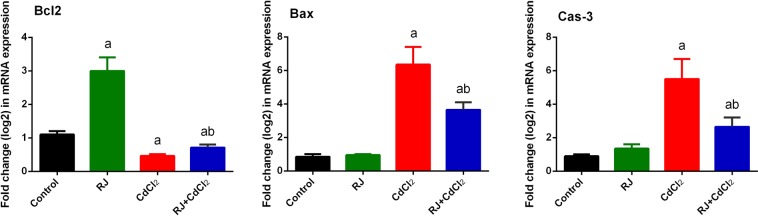
Effects of royal jelly (RJ) on the gene expressions of Bcl-2, Bax, and caspases-3 in the kidney of CdCl_2_-treated mice. Data (mean ± SD of triplicate assays) were normalized to those of GAPDH and expressed as fold induction (log 2 scale), relative to the mRNA level in the control. ^a^*p* < 0.05 *vs*. the control mice; ^b^*p* < 0.05 *vs*. the CdCl_2_-treated mice analyzed using Tukey’s test.

In the kidney CdCl_2_-treated mice, Bax and caspase 3 immunoreactivities were stronger than those of the control mice (Fig. 12), whereas the immunoreactivity of Bcl-2 was weaker than that of the control mice. Interestingly, RJ pretreatment markedly reduced the expression of Bax and caspase-3, and markedly increased Bcl-2 expression compared with the Cd-treated group (Table 1). In consistence with the immunohistochemistry findings, qRT-PCR results showed that the mRNA expression levels of *Bax* and *casp3* were significantly higher in the kidney of CdCl_2_-treated mice, whereas *Bcl2* expression was diminished. However, Cd-induced apoptosis in the kidney was blocked by RJ pretreatment (Fig. 13).

**Table 1:** Effects of royal jelly on the immunohistochemistry intensity of Bcl-2, Bax and Caspase 3 in renal tissue of mice treated Cd-induced nephrotoxicity.

Apoptosis protein	Control	Royal jelly	Cadmium chloride	Royal jelly + Cadmium chloride
Bcl-2	++	+++	+	++
Bax	+	+	+++	++
Caspase 3	+	+	+++	++

Note: + = Weak immunoreactivity, ++ = Moderate immunoreactivity, +++ = Strong immunoreactivity and ++++ = Very strong immunoreactivity.

## Discussion

Cadmium is considered as the most common environmental and accidental pollutant in developing countries that often leads to a serious threat to human and animals^22^. Cadmium accumulates mainly in metabolically active tissues, such as in the kidney. Repeated exposure to Cd leads to organ and systemic injuries, particularly the kidneys^2^. In the liver, Cd binds to metallothionein, a cysteine-rich protein with sulfhydryl group, to form Cd-MT complex. This complex reaches the kidney through circulation, where it is filtered through the glomeruli and reabsorbed by divalent metal-ion transporter-1 in the proximal tubular epithelial cells, in which proteases in the lysosomes degraded the complex to release Cd. However, the released Cd rebinds to newly synthesized MT, which causes kidney damage^4^. However, after the MT binding capacity is saturated, a large amount of Cd ion is liberated into the cytosol and Cd can directly affect and damage the integrity of plasma membranes and intracellular organelles causing cell death^23,24^. Accordingly, kidney concentrations of cadmium and metallothionein in Cd-treated mice in the current study were increased significantly, whereas the absolute kidney weight was decreased significantly. Kidney weight is presented either as absolute or relative (kidney to body) weight. In general, organ mass is balanced between differentiation, proliferation, and death, and a shift towards apoptosis in the equilibrium may lead to a reduction in organ mass. Therefore, the reported effect of Cd on kidney weights in the this study may be caused by Cd-induced oxidative injury in the kidney tissue^25^. However, Cd concentration and absolute kidney weight were maintained near normal level by RJ pretreatment as compared with those of Cd-treated mice. This suggested potential metal chelating and antioxidant activities of RJ^26^. The RJ chelation property may be due to the ability of RJ to (a) enhance MT synthesis and (b) facilitate Cd-MT execration. However, the ability of RJ to facilitate Cd removal from the kidney in the present study is still unclear and needs further study.

In the present study, Cd intoxication increased KIM-1 protein in the kidney tissue. Whilst, RJ pretreatment negated this. KIM-1 is a type I transmembrane protein molecule that is not detectable in healthy renal tissue, but is upregulated in de-differentiated proximal tubule epithelial cells after ischemic or toxic injury. KIM-1 is an early marker of Cd-induced proximal tubular injury^27^.

In the current study, increased level of kidney function markers reflected the deterioration of kidney function following Cd injection in mice. Kidney is vulnerable to cadmium toxicity because it receives 20% of cardiac output containing high amount excreted substances, which may accumulate in the tubular cells. Cd accumulation in the kidney leads to reduced glomerular filtration rate, polyuria, and severe tubular dysfunction. The decrease in glomerular filtration rate leads to accumulation of endogenous wastes, toxicants, and xenobiotics^28^. In consistence with the findings of the current study, histopathology of kidney tissue includes shrinkage and degradation of glomerulus, severe degradation of the tubular lining cells, and vascular congestion. In the present study, histopathological examinations showed that RJ pretreatment led to almost complete restoration of the CdCl_2_-induced renal damage and prevention against Cd-induced elevation in the level of kidney function markers. These results were consistent with those of Karadeniz *et al*.^29^, in which RJ restored kidney structure and function following cisplatin treatment.

Cadmium-induced kidney dysfunction in our study was most likely the consequence of oxidant/antioxidant imbalance in the kidney, since it was parallel with elevated LPO and NO levels, which could be regarded as signals of increased generation of reactive oxygen species (ROS) and reactive nitrogen species (RNS). The reductions of enzymatic and non-enzymatic antioxidant molecules in the renal tissue of cadmium-exposed mice also supported these findings, suggesting that antioxidant defense system was unable to cope with the increase of ROS/RNS that resulted in cellular damage and death. In the current study, Cd intoxication caused lipid peroxidation that led to impairment of the structural and functional integrity of cell membrane. Furthermore, the increase in lipid peroxidation in the renal tissue causes the overconsumption and depletion of functional thiol (-SH) groups in several enzymatic and non-enzymatic molecules^30^. Cadmium injection increased iNOS expression, which led to more NO production. The deleterious effect of NO is observed once it reacts with superoxide anion, generating exceedingly reactive peroxynitrite anion (ONOO−). NO also has been known to exert pro-apoptotic effects via the c-Jun N-terminal kinase signaling pathway and caspases 3 and 6^31^. However, pretreatment with RJ markedly restored the oxidative stress markers (LPO and NO) near the normal levels. This finding was in line with that of Ahmed *et al*.^32^, which revealed that RJ is able to reverse all deleterious effects of Cd.

The present study also showed a decrease in glutathione content and enzymatic activity of antioxidants, such as SOD, GSH-Px, CAT, and GSH-R, in renal tissue of CdCl_2_-injected mice. In the absence of cadmium, endogenous antioxidants are important scavenger of ROS. However, cadmium showed a high affinity for binding to sulfhydryl group, which is associated with the depletion of endogenous sulfhydryl compounds^4^. Cd-induced oxidative damage, which is further supported by the increase in LPO and NO levels as well as the downregulation of antioxidant and stress response genes, could therefore be associated with insufficient antioxidant activity and eventual cellular dysfunction and death^33^. Inhibition in the activities of SOD, CAT, GSH-Px, and GSH-R in the renal tissue of CdCl_2_-injected mice could be caused by the reduced synthesis of enzymes or oxidative inactivation of protein enzymes^22^. Our findings in this study were in agreement with other studies, which revealed that cadmium induced kidney toxicity by enhancing ROS formation, GSH consumption, and inhibiting antioxidant-mediated defense system^34^. However, this study also suggested that the pre-administration of RJ prior to cadmium injection prevented the alteration in enzymatic and non-enzymatic activity of antioxidant molecules, and the antioxidant and metal-chelating properties of RJ also prevented the generation of free radicals, further reducing the cadmium-induced oxidative damage. Furthermore, RJ contains free amino acids such as methionine, proline, cysteine and cystine might responsible for this effect by promoting biosynthesis of glutathione and scavenging free radicals, in addition to the ability of RJ to enhance the activity of antioxidant enzymes^35^.

In the current study, we found that CdCl_2_ injection induced the downregulation of Nrf2 expression in mice kidney. Nrf2 is a transcription factor that ameliorates in injury- and inflammation-induced damage by regulating the expression of antioxidant genes by binding to the antioxidant response element (ARE) in the upstream promoter region of many antioxidant genes like NQO1 and HO-1, initiating their transcription. However, Montes *et al*.^36^ found that Cd activates Nrf2 in rat kidney as a compensatory mechanism of oxidative stress to counteract the toxic effects of Cd and prevent apoptosis. In our present study, RJ pretreatment reversed this effect, indicating that RJ may be a potent antioxidant against Cd-induced nephrotoxicity by modulating the expression of antioxidant and detoxification enzymes.

Additionally, inflammation may complicate the pathologic processes of oxidative stress. In the current study, based on the elevated levels of IL-1β and TNF-α, Cd intoxication in mice caused inflammatory response. TNF-α is a cell signaling cytokine involved in systemic inflammation by firing up the acute phase reaction. TNF-α can activate three different pathways, namely mitogen-activated protein kinase (MAPK), NF-κB, and cell death signaling pathways. Mehaffey and Majid *et al*.^37^ found that TNF-α can promotes kidney dysfunction by direct cytotoxicity, vasoconstriction, and promoting inflammatory cells infiltration, which deteriorate both tubular function and viability. Wongmekiat *et al*.^38^ also showed that Cd-induced nephrotoxicity is associated with TNF-α. IL-1β is also an important mediator of the inflammatory response involved in cell proliferation, differentiation, and apoptosis^39^. Odewumi *et al*.^40^ found that Cd highly upregulated IL-1β. In the present study, the increases in TNF-α and IL-1β were regressed by RJ pretreatment, which were consistent with previous findings of Chen *et al*.^41^, in which RJ exhibited a strong inhibitory effect on Nos2, *Il1β*, and *Tnf* mRNA generation by suppressing the activity of NF-κB.

Bcl-2, Bax, and caspase 3 are involved in the mitochondria-mediated intrinsic apoptosis pathway. Bcl-2, located in the outer membrane of mitochondria, plays a pivotal task in inhibiting the actions of pro-apoptotic proteins and enhancing cellular survival. Bax is a cytosolic protein that increases the opening of the mitochondrial anion channel, which leads to the release of cytochrome c and loss of mitochondrial membrane potential. Caspase 3 is the final protease that activates apoptotic DNA fragmentation^21^. In the current study, immunohistochemistry analysis showed that Bax and caspase 3 expressions in Cd-treated mice kidney were markedly elevated, but Bcl-2 expression was decreased, suggesting that Cd initiated the mitochondria-mediated apoptotic pathway. These findings were consistent with those of Bao *et al*.^42^, in which chicken kidney exposed to Cd showed decreased Bcl-2 and increased Bax and caspase 3 levels. However, RJ pretreatment effectively reversed this Cd-induced process, suggesting that RJ resisted apoptosis by enhancing the expression level of anti-apoptotic protein Bcl-2. This finding was in accordance with that of Amiri *et al*.^43^, which found that RJ increased the expression of Bcl-2, but not Bax in mature oocytes, improved blastocyst formation, and decreased the apoptotic incidence during the *in vitro* development of sheep cumulus cells.

## Conclusion

Our study evidently showed that RJ pretreatment exerted protective effect against Cd-induced nephrotoxicity in mice by facilitating Cd execration, restoring the oxidant/antioxidant balance and preventing inflammation and apoptosis. RJ could be a potent agent for renal protection against Cd-induced nephrotoxicity. Further studies on the Cd removal mechanism by RJ must be conducted to elucidate the main mechanism that responsible for RJ nephroprotective effect. The current study will provide a direction and basis for such further studies.

## Supplementary information

Supplement
