# Supplementary material for: Royal jelly attenuates cadmium-induced nephrotoxicity in male mice
Source: Sci Rep. 2019 Apr 9;9:5825. doi: 10.1038/s41598-019-42368-7 (PMC6456607; doi:10.1038/s41598-019-42368-7)

Royal jelly attenuates cadmium-induced nephrotoxicity in male mice

Rafa S. Almeer[1,*], Gadah I. AlBasher[1], Saud Alarifi[1], Saad Alkahtani[1], Daoud Ali[1] and Ahmed E. Abdel Moneim[2]

[1] Department of Zoology, College of Science, King Saud University, Riyadh, Saudi Arabia

[2] Department of Zoology and Entomology, Faculty of Science, Helwan University, Cairo, Egypt

Supplementary Table 1. Primer sequences of genes analyzed in real time PCR

Name	Accession number	Forward primer (5'---3')	Reverse primer (5'---3')
Gapdh	NM_017008.4	AGTGCCAGCCTCGTCTCATA	GATGGTGATGGGTTTCCCGT
Sod2	NM_017051.2	TAAGGGTGGTGGAGAACCCA	TGATGACAGTGACAGCGTCC
Cat	NM_012520.2	TTTTCACCGACGAGATGGCA	AAGGTGTGTGAGCCATAGCC
Gpx1	NM_030826.4	CAGTCCACCGTGTATGCCTT	GTAAAGAGCGGGTGAGCCTT
Gsr	NM_053906.2	TACTGCACTTCCCGGTAGGA	TGGATGCCAACCACCTTCTC
Nfe2l2	NM_031789.2	TTGTAGATGACCATGAGTCGC	ACTTCCAGGGGCACTGTCTA
Nos2	NM_012611.3	GTTCCTCAGGCTTGGGTCTT	TGGGGGAACACAGTAATGGC
Il1β	NM_031512.2	GACTTCACCATGGAACCCGT	GGAGACTGCCCATTCTCGAC
TNF-α	NM_012675.3	GGCTTTCGGAACTCACTGGA	CCCGTAGGGCGATTACAGTC
Bcl2	NM_016993	ACTCTTCAGGGATGGGGTGA	TGACATCTCCCTGTTGACGC
Bax	NM_017059.2	GGGCCTTTTTGCTACAGGGT	TTCTTGGTGGATGCGTCCTG
Casp3	NM_012922.2	GAGCTTGGAACGCGAAGAAA	TAACCGGGTGCGGTAGAGTA

The abbreviations of the genes; G*apdh*, glyceraldehyde-3-phosphate dehydrogenase; *Sod2*, superoxide dismutase 2 mitochondrial (MnSOD); *Cat*, catalase; *Gpx1*, glutathione peroxidase 1; *Gsr*, glutathione reductase; *Nfe2l2*, nuclear factor erythroid 2-related factor 2; *Nos2*, inducible nitric oxide synthase; *Il1β*, interleukin 1 beta; *Tnf*, tumor necrosis factor; *Bcl2*: B-cell lymphoma 2; *Bax*, Bcl-2-like protein 4; *Casp3*, caspase-3.

Supplementary Figure S1: Full-length blots/gels of different proteins.

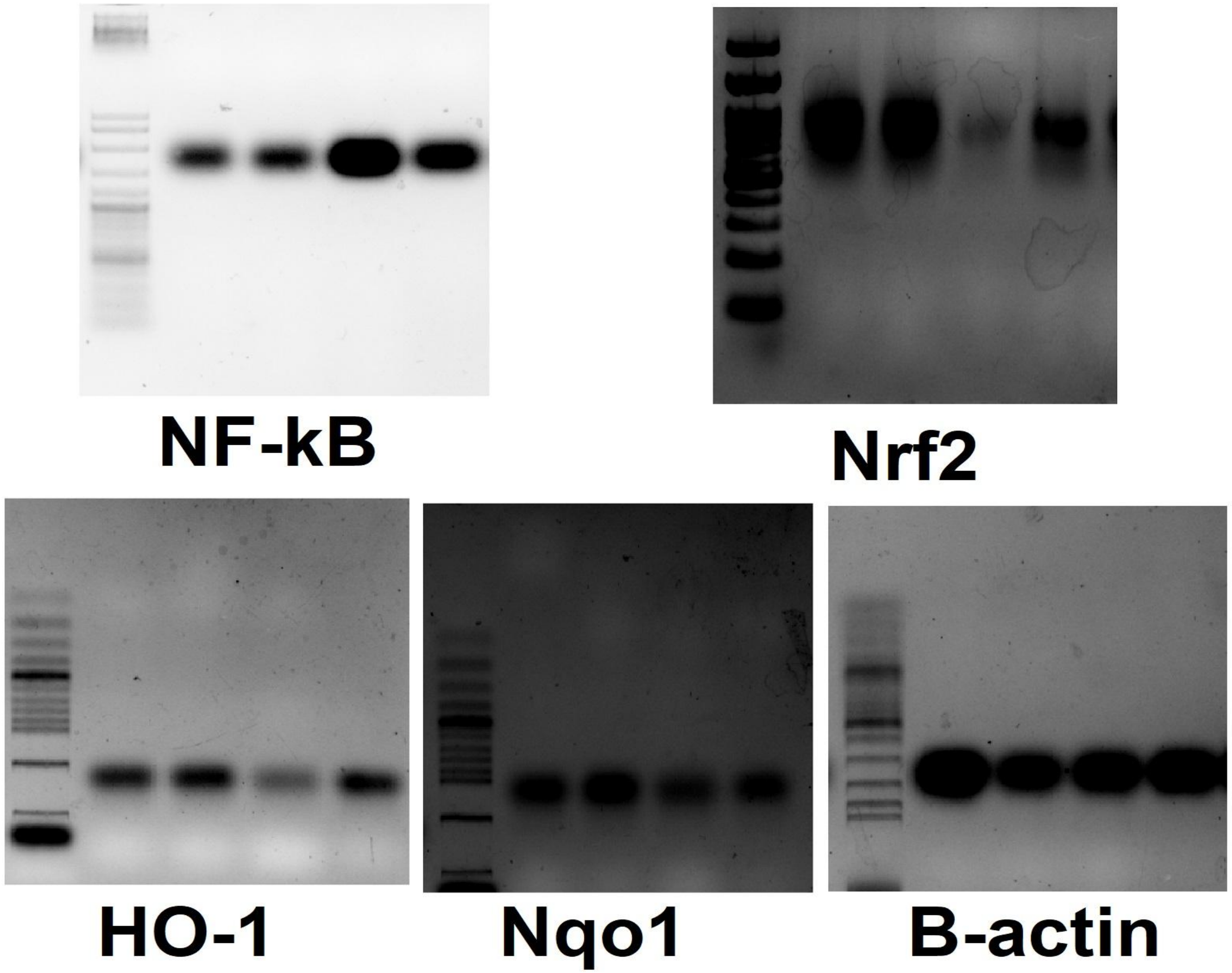

Supplement: Supplementary file 1 — Supplement [file 41598_2019_42368_MOESM1_ESM.pdf]
